# Electrospun PVA Nanofibers Co-Loaded with Atorvastatin and Zinc Oxide for Antibacterial and In Vitro Wound Healing Applications

**DOI:** 10.3390/biomedicines14030724

**Published:** 2026-03-20

**Authors:** Rawan Fitaihi, Alanoud Altalal, Rihaf Alfaraj, Fai Alkathiri, Riyad F. Alzhrani, Shumukh Aldawsari, Shouq Alorayyidh, Meshal Alnefaie, Nojoud Al Fayez, Njoud Altuwaijri

**Affiliations:** 1 Department of Pharmaceutics, College of Pharmacy, King Saud University, Riyadh 11451, Saudi Arabiaralfaraj@ksu.edu.sa (R.A.); falkathire@ksu.edu.sa (F.A.); rfalzahrani@ksu.edu.sa (R.F.A.);; 2 Advanced Diagnostics and Therapeutics Institute, Health Sector, King Abdulaziz City for Science and Technology (KACST), Riyadh 11442, Saudi Arabia; malnefaie@kacst.gov.sa (M.A.);

**Keywords:** electrospun nanofibers, atorvastatin, zinc oxide nanoparticles, antibacterial activity, Gram-positive bacteria, methicillin-resistant *Staphylococcus aureus* (MRSA), in vitro wound healing, scratch assay

## Abstract

**Background:** The global rise in antimicrobial resistance (AMR) has created an urgent need for innovative antibacterial strategies and localized delivery systems. This study aimed to develop and characterize electrospun poly (vinyl alcohol) (PVA) nanofibers co-loaded with atorvastatin (ATR) and zinc oxide (ZnO) nanoparticles for use as a multifunctional topical platform for wound healing and infection control. **Methods:** ZnO nanoparticles were prepared via ball milling and characterized for size and zeta potential. Four PVA-based nanofiber formulations were fabricated using electrospinning: blank (F1), ZnO-loaded (F2), ATR-loaded (F3), and ATR/ZnO co-loaded (F4). The nanofibers were evaluated for morphology, thermal properties, crystallinity, and drug release. Antibacterial efficacy was tested against *S. aureus*, *S. epidermidis*, MRSA, and *P. aeruginosa* using broth microdilution and checkerboard assays. Biocompatibility and wound healing potential were assessed via MTT and fibroblast scratch assays on human foreskin fibroblasts (hFFs). **Results:** SEM imaging confirmed the production of uniform, bead-free nanofibers. ATR and ZnO nanoparticles were successfully incorporated in the nanofiber. The co-loaded formulation (F4) demonstrated a sustained release profile, releasing approximately 78.7% of ATR over 24 h. While all treatments showed limited activity against *P. aeruginosa*, the ATR/ZnO co-loaded nanofibers exhibited significantly enhanced antibacterial activity against Gram-positive strains, achieving the lowest MIC values (1.5–2.0 mg/mL). Synergy analysis confirmed an enhanced effect with ATR and ZnO against MRSA. Furthermore, F4 achieved the highest wound closure rate of 92.41% in 24 h while maintaining acceptable cytocompatibility. **Conclusions:** The integration of ATR and ZnO into PVA nanofibers provides an enhanced antibacterial effect consistent with the synergistic potential observed between free agents targeting Gram-positive wound pathogens. The platform’s ability to simultaneously inhibit bacterial growth and promote rapid fibroblast migration positions it as a promising localized therapeutic for managing infected wounds.

## 1. Introduction

Topical antimicrobial therapy plays a pivotal role in the management of skin infections and prevention of wound-related complications, especially in an era marked by the global rise in antimicrobial resistance (AMR). Traditional topical formulations such as creams, gels, and ointments are widely used; however, their limited retention, burst drug release, and suboptimal penetration can hinder therapeutic efficacy in localized infections. In response, innovative drug delivery systems have been explored to enhance local drug bioavailability, reduce systemic side effects, and improve treatment outcomes in infected or compromised skin tissues [1].

Drug repurposing is a promising strategy in which existing medications are explored for new therapeutic indications. Atorvastatin (ATR), a widely prescribed statin that inhibits 3-hydroxy-3-methylglutaryl coenzyme A (HMG-CoA) reductase, the key enzyme in cholesterol biosynthesis, has gained significant attention for its pleiotropic properties beyond lipid-lowering. ATR has been shown to exhibit antimicrobial, anti-inflammatory, angiogenic, and antioxidant effects, making it an attractive candidate for repurposing in topical antibacterial therapies. Its antibacterial action is attributed to membrane disruption, inhibition of bacterial metabolism, suppression of quorum sensing, and interference with biofilm formation [2]. Studies have reported its effectiveness against *Staphylococcus aureus* (*S. aureus*), *Escherichia coli*, and *Candida albicans* [3,4,5,6,7]. Furthermore, encapsulating ATR in suitable delivery platforms has been demonstrated to enhance its solubility, protect against degradation, and improve site-specific delivery [8,9].

Among the advanced delivery systems, electrospun nanofibers have emerged as a versatile platform for topical and transdermal drug delivery. Electrospinning allows for the fabrication of fibrous mats with nanoscale diameters, mimicking the structural and functional characteristics of the extracellular matrix (ECM). The high surface area-to-volume ratio, tunable porosity, and flexibility in incorporating hydrophilic and hydrophobic drugs make electrospun fibers highly suitable for localized delivery in skin applications [10]. Moreover, their capacity to provide sustained drug release, absorb wound exudates, and act as physical barriers against microbial invasion has established their utility in therapeutic dressing technologies [11,12]. Recent advances in multifunctional wound-healing nanocomposites based on polyvinyl alcohol (PVA), alginate, hyaluronic acid, and chitosan matrices incorporating bioactive nanoparticles further highlight the growing interest in polymer–nanoparticle systems for simultaneous antibacterial protection and tissue regeneration [13,14,15].

Incorporating antibacterial agents into nanofibers not only enhances local bioactivity but can also reduce the risk of systemic toxicity. In this regard, zinc oxide (ZnO) nanoparticles have gained considerable interest as inorganic antibacterial agents. Recognized as Generally Regarded As Safe (GRAS) by the FDA, ZnO exhibits strong antibacterial activity through multiple mechanisms, including the generation of reactive oxygen species (ROS), release of Zn^2+^ ions, and disruption of microbial cell membranes [16,17]. Additionally, ZnO has been reported to promote tissue regeneration through angiogenesis stimulation and epithelialization, thereby serving dual roles in both infection control and tissue repair [18,19]. While ATR antibacterial and host-modulating effects have been observed in systemic therapeutic combinations [20] and ZnO nanoparticles are widely recognized for their antibacterial and wound-healing potential [21], their co-delivery within an electrospun nanofiber system that provides localized antibacterial activity and wound support has not been previously reported, positioning the current work as a novel multifunctional topical antibacterial and therapeutic platform.

Recent studies suggest that combining ZnO nanoparticles with various therapeutics, could result in synergistic effects, enhancing antibacterial efficacy through complementary mechanisms while minimizing the required dose of each component [22,23,24].

Despite the individual advantages of ATR and ZnO, their co-delivery using electrospun nanofiber systems remains largely unexplored. The integration of both agents into a single fibrous matrix has the potential to address multiple challenges associated with skin infections, namely, microbial colonization, inflammation, and suboptimal drug penetration, offering a tailored and multi-mechanistic strategy for topical antibacterial therapy.

This study aims to develop and characterize electrospun nanofibrous mats co-loaded with ATR and ZnO nanoparticles (ZnO NPs) for potential applications as a topical antibacterial system. The formulation strategy focuses on enhancing the bioavailability and sustained delivery of ATR, while leveraging the broad-spectrum antibacterial properties of ZnO.

## 2. Materials

PVA 98% hydrolyzed with viscosity (25–32 cps) was purchased from Loba Chemie (Mumbai, India). Pure ATR was provided by Spimaco Addwaeih (Riyadh, Saudi Arabia). ZnO was acquired from Avonchem (Cheshire, UK). Dimethyl sulfoxide (DMSO), methanol, and acetonitrile (ACN) (HPLC grade) were all purchased from Sigma Aldrich (St. Louis, MO, USA).

Mueller–Hinton broth (MHB) was purchased from Oxoid (Basingstoke, UK). All reagents were of analytical grade. The antibacterial activity of free drugs and nanofiber formulations was evaluated against the following bacterial strains obtained from American Type Culture Collection (ATCC, Manassas, VA, USA): *Staphylococcus aureus* (*S. aureus*) ATCC 25923, *Staphylococcus epidermidis* (*S. epidermidis*) ATCC 12228, methicillin-resistant *Staphylococcus aureus* (MRSA) ATCC 43300, and *Pseudomonas aeruginosa* (*P. aeruginosa*) ATCC 27853. All strains were stored at −80 °C in glycerol stocks and subcultured on agar media prior to use in antibacterial assays.

Human foreskin fibroblasts (hFFs) were obtained from ATCC SCRC-1041™ (Manassas, VA, USA). Cells were cultured according to the supplier’s instructions and used at passages 7–10. Dulbecco’s Modified Eagle Medium (DMEM), fetal bovine serum (FBS), penicillin–streptomycin solution, phosphate-buffered saline (PBS), and trypsin–EDTA (0.25%) were purchased from Thermo Fisher Scientific (Gibco™, Waltham, MA, USA). 3-(4,5-dimethylthiazol-2-yl)-2,5-diphenyltetrazolium bromide (MTT) was purchased from Thermo Fisher Scientific (Gibco™, Waltham, MA, USA). All reagents were of cell-culture grade and used as received.

## 3. Methods

### 3.1. Preparation of Nanosized ZnO

ZnO NPs were prepared via top-down milling using a planetary ball mill (Pulverisette 7, Fritsch GmbH, Idar-Oberstein, Germany). The milling process was conducted at a rotational speed of 1000 rpm in three repeated cycles, with each cycle consisting of 10 min of milling followed by a 5 min pause to prevent overheating. The milling mixture comprises 1 g of ZnO powder, 90 g of zirconia milling balls, and 25 mL of a 3% *w*/*v* PVA aqueous solution, which serves as a stabilizing agent to prevent particle agglomeration. The resulting dispersion was collected as a ZnO nanosuspension.

### 3.2. Characterization of ZnO Nanosuspension

The average particle size, polydispersity index (PDI), and surface charge (zeta potential) of the prepared ZnO nanosuspension were measured using dynamic light scattering (DLS) and electrophoretic light scattering, respectively, with a Zetasizer Nano ZS (Malvern Instruments Ltd., Malvern, UK). Samples were appropriately diluted with deionized water prior to measurement to avoid multiple scattering effects. The concentration of ZnO NPs in the nanosuspension was determined gravimetrically by drying a known volume of the dispersion in a pre-weighed crucible at 40 °C until a constant weight was achieved. The final concentration was calculated based on the mass of the dried residue.

### 3.3. Preparation of Electrospun Nanofibers

Four different electrospun formulations were prepared, and their compositions are presented in Table 1. The ATR and ZnO nanoparticle loadings were selected to ensure stable electrospinning and the formation of uniform, bead-free fibers while providing sufficient active content for antibacterial evaluation. These loading levels were determined through preliminary optimization to balance electrospinnability, fiber morphology, and antibacterial effectiveness while avoiding bead formation or jet instability at higher concentrations. Electrospinning of all formulations was conducted under controlled ambient conditions. The spinning solutions were prepared to achieve a total solids concentration of 10% (*w*/*v*) relative to the solvent system, and the tip-to-collector distance was consistently maintained at 20 cm for every formulation to ensure comparability of fiber formation. Minor adjustments in applied voltage and needle diameter were required to obtain a stable Taylor cone and continuous jet, reflecting formulation-dependent differences in solution viscosity and electrical conductivity following incorporation of ATR and/or ZnO nanoparticles [25].

To prepare F1, a blank PVA solution was obtained by dissolving PVA in 9.5 mL of deionized water, followed by the addition of 0.5 mL DMSO and 1 mL methanol. The mixture was stirred until a homogeneous solution was formed, loaded into a 10 mL syringe fitted with a size 7 needle, and electrospun at an applied voltage of 13–14 kV and a flow rate of 1.0 mL/h.

For F2 (ZnO-loaded fibers), PVA was dissolved in 7.5 mL of deionized water, after which the ZnO nanosuspension was added and the mixture was stirred thoroughly. The resulting dispersion was electrospun using a size 8 needle, an applied voltage of 17–18 kV, and a flow rate of 1.0 mL/h.

To prepare F3 (ATR-loaded nanofibers), ATR was first dissolved in a solvent mixture of 0.5 mL DMSO and 1 mL methanol, while PVA was dissolved separately in 9 mL of water. The two solutions were then combined, stirred, and loaded into a 10 mL syringe fitted with a size 8 needle. Electrospinning was performed at an applied voltage of 18–19 kV, a flow rate of 1.0 mL/h.

The composite formulation containing both ATR and ZnO NPs (F4) was prepared by dissolving ATR in 0.5 mL DMSO, followed by the addition of 1 mL methanol, while PVA was dissolved separately in water. After thorough mixing of the ATR and PVA solutions, the ZnO nanosuspension was incorporated into the blend. The final formulation was electrospun using a size 8 needle, an applied voltage of 17–18 kV, and a flow rate of 1.1 mL/h.

### 3.4. Characterization of Nanofibers

#### 3.4.1. Nanofiber Morphology and Size Analysis

The surface morphology and diameter of the blank and drug-loaded electrospun nanofibers were examined using a scanning electron microscope (SEM) (JSM-IT500HR, JEOL Inc., Peabody, MA, USA) operated at an accelerating voltage of 5 kV. Prior to imaging, the nanofiber mats were mounted on SEM stubs and sputter-coated with a 2 nm platinum layer using an auto fine coater (JEC-3000FC, JEOL Inc.) to enhance conductivity. For each formulation, fiber diameter was measured from SEM images using ImageJ 1.54g software (National Institutes of Health, Bethesda, MD, USA), with a minimum of 50 individual fibers analyzed per sample to calculate the average diameter and distribution.

#### 3.4.2. Thermal Analysis

The thermal behavior of the nanofibers was assessed using Differential Scanning Calorimetry (DSC) DSC 4000, PerkinElmer (Shelton, CT, USA). Approximately 5 mg of each sample was weighed into a standard aluminum pan and hermetically sealed, with an empty pan used as a reference. Samples were heated from 30 °C to 400 °C at a rate of 10 °C/min under a nitrogen atmosphere flowing at 40 mL/min. The temperature was held at 30 °C for 1 min prior to heating.

#### 3.4.3. Infrared Spectroscopy

Fourier-Transform Infrared Spectroscopy (FTIR) analysis was conducted to evaluate potential interactions between components and confirm drug incorporation into the fibers. Spectra were recorded using a Thermo Smart ATR IS20 spectrometer (Thermo Fisher Scientific, Waltham, MA, USA) with a resolution of 4 cm^−1^ and 32 scans per sample. Approximately 7 mg of each sample was placed directly on the ATR crystal and scanned over the spectral range of 4000–650 cm^−1^.

#### 3.4.4. X-Ray Analysis

X-ray diffraction (XRD) analysis was performed to investigate the crystallinity of the nanofibers. Measurements were carried out using a Rigaku Miniflex 300/600 diffractometer (Tokyo, Japan) equipped with a Cu Kα radiation source, operated at 40 kV and 15 mA. Samples were mounted on glass holders and scanned over a 2θ range of 3° to 50° at a scanning rate of 5°/min.

### 3.5. Drug Loading and Entrapment Efficiency

To quantify the amount of ATR incorporated into the nanofibers, both ATR-only and ATR-ZnO composite formulations were analyzed. Multiple nanofiber samples (*n* ≥ 3) were collected from different regions of the electrospun mats and from two batches to ensure representative evaluation of drug distribution. A known mass (5 mg) of each fiber sample was accurately weighed and dissolved in 5 mL of a 1:1 (*v*/*v*) ethanol–water mixture. The solutions were incubated under gentle shaking at 37 °C until complete dissolution was achieved, then filtered to remove any undissolved particulates. The filtrates were analyzed using high-performance liquid chromatography (HPLC) to determine the ATR content.

The loaded nanofiber was analyzed with a Waters e2695 HPLC system, which included a Waters^®^ 717 plus autosampler, a Waters 600 binary pump, and a Waters 2489 UV detector (Waters Corporation, Milford, MA, USA). The separation was accomplished using gradient elution with a mobile phase containing 0.1% phosphoric acid (A)and acetonitrile (B). A linear gradient was conducted at 1 mL/min with 95% solvent A and 5% solvent B at the start. Increasing to 95% B from 2 to 12 min. Maintain the same ratio until t = 17 min. The gradient was then restored to 95% solvent A and 5% solvent B at 18 min before being stabilized for 20 min. The chromatographic analysis was performed using an XBridge C18 column (5 µm, 4.6 × 250 mm; Waters Corporation, Milford, MA, USA) maintained at 20 °C, with a 10 µL injection volume, and detection at 240 nm.

Drug loading (*DL*%) and entrapment efficiency (*EE*%) were calculated using the following equations [26]:

$$DL\;\left(\%\right)=\frac{\mathrm{Amount}\;\mathrm{of}\;\mathrm{drug}\;\mathrm{encapsulated}}{\mathrm{Total}\;\mathrm{weight}\;\mathrm{of}\;\mathrm{nanofibe}}\times 100$$

$$EE\left(\%\right)=\frac{\mathrm{Amount}\;\mathrm{of}\;\mathrm{drug}\;\mathrm{encapsulated}}{\mathrm{Initial}\;\mathrm{amount}\;\mathrm{of}\;\mathrm{drug}\;\mathrm{used}}\times 100$$

### 3.6. In Vitro Drug Release Study

The in vitro release profile of ATR from the electrospun nanofibers was evaluated under physiological temperature conditions. Approximately 10 mg of each fiber sample (F3 and F4) was placed in 5 mL of a dissolution medium consisting of ethanol and phosphate-buffered saline (PBS) (pH 7.4) in a 2:3 (*v*/*v*) ratio. The samples were incubated in a temperature-controlled water bath shaker set at 37 °C with continuous gentle agitation.

At predetermined time intervals, 15, 30, 60, 90, 120, 180, 240, 300 min, and 24 h, aliquots of the release medium were withdrawn for analysis and immediately replaced with an equal volume of fresh pre-warmed dissolution medium to maintain sink conditions and constant volume. The collected samples were filtered, and the concentration of ATR was quantified using HPLC.

### 3.7. Antibacterial Activity

#### 3.7.1. Bacterial Strains and Inoculum Preparation

Antibacterial activity was evaluated against *S. aureus*, *S. epidermidis*, MRSA, and *P. aeruginosa*. Bacterial cultures were grown overnight at 37 °C and adjusted to a 0.5 McFarland standard, followed by dilution in MHB to obtain a final inoculum of approximately 5 × 10^5^ CFU/mL.

#### 3.7.2. Preparation of Free Drug Solutions

ATR stock solutions were prepared and diluted in DMSO such that the final solvent concentration did not exceed 1% (*v*/*v*). ZnO was prepared from a stabilized suspension and sonicated prior to dilution to ensure uniform dispersion. Free ATR, ZnO, and ATR/ZnO combinations were tested using concentration ratios consistent with the ATR:ZnO loading ratio in the nanofiber formulations, employing two-fold serial dilutions.

#### 3.7.3. Broth Microdilution Assay

The antibacterial activity of nanofiber formulations and free agents was evaluated using a broth microdilution assay adapted for solid formulations [27]. Four nanofiber formulations were tested (F1–F4) alongside free ATR and ZnO.

Nanofiber extractions were done by incubating 4 mg of nanofibers in 1.0 mL of medium at 37 °C for 24 h under gentle agitation. Following incubation, suspensions were centrifuged at 10,000× *g* for 5 min to remove residual fibers, and the supernatants were collected. These extracts were considered to represent a 4 mg/mL nanofiber-equivalent stock solution.

MICs were determined using sterile 96-well microplates, with each well containing a final volume of 200 µL (100 µL of test solution and 100 µL of bacterial suspension). Plates were incubated at 37 °C for 24 h under aerobic conditions.

#### 3.7.4. Bacterial Growth Assessment

Bacterial growth was assessed by measuring optical density at 600 nm after 24 h incubation. Blank-subtracted optical density values (OD_net_) were calculated to correct any background turbidity.

#### 3.7.5. Checkerboard Combination Assay and Synergy Analysis

Synergistic interactions between ATR and ZnO NPs were evaluated using an 8 × 8 checkerboard microdilution assay. Two-fold serial dilutions of ATR were combined with two-fold serial dilutions of ZnO NPs and incubated at 37 °C for 24 h.

Synergy was quantified using the fractional inhibitory concentration index (*FICI*) [28]:

$$FICI=\frac{MIC_{\mathrm{ATR}\;\mathrm{in}\;\mathrm{combination}}}{MIC_{\mathrm{ATR}}}+\frac{MIC_{\mathrm{ZnO}\;\mathrm{in}\;\mathrm{combination}}}{MIC_{\mathrm{ZnO}}}$$

Interactions were interpreted as synergistic (*FICI* ≤ 0.5), additive (0.5 < *FICI* ≤ 1.0), indifferent (1.0 < *FICI* ≤ 4.0), or antagonistic (*FICI* > 4.0). All experiments were performed in triplicate, and data are presented as mean ± SD.

### 3.8. In Vitro Cytotoxicity Assay

The cytocompatibility of the electrospun nanofiber formulations (F1–F4) was evaluated using an extract-based MTT assay on human foreskin fibroblasts (hFFs). Sterilized nanofiber samples were incubated in complete DMEM at a concentration of 4 mg/mL and maintained at 37 °C for 24 h to obtain material extracts. The collected extracts were subsequently diluted with fresh culture medium to obtain final concentrations of 2 mg/mL and 1 mg/mL.

The hFFs were seeded in 96-well plates at a density of 5 × 10^3^ cells per well and allowed to attach for 24 h under standard culture conditions (37 °C, 5% CO_2_). The culture medium was then replaced with the prepared nanofiber extracts, and cells were incubated for an additional 24 h. Cells cultured in fresh medium served as the negative control. After exposure, cell viability was assessed using an MTT assay. Briefly, MTT solution (0.5 mg/mL) was added to each well and incubated for 4 h. The resulting formazan crystals were dissolved in DMSO, and absorbance was measured at 570 nm using a microplate reader (BioTek Synergy™, Winooski, VT, USA). Cell viability was calculated as a percentage relative to the untreated control group.

### 3.9. In Vitro Wound Healing Activity

The hFFs cells were seeded at a density of 5 × 10^5^ cells/mL into 12-well plates and cultured in DMEM supplemented with 10% FBS and 1% penicillin-streptomycin. The cells were incubated at 37 °C in a humidified atmosphere of 5% CO_2_ until a uniform confluent monolayer was established. To simulate a wound, a straight linear scratch was carefully made at the center of each well using a sterile 100 μL pipette tip. The culture medium was then aspirated, and the wells were gently rinsed with PBS to remove detached cells and debris. Fresh DMEM was added to each well, after which UV-sterilized nanofiber mats corresponding to each formulation (F1–F4) were placed into the wells, the fiber was allowed to freely interface with the culture medium. Untreated wells incubated with fresh medium alone served as the negative control. All treatments were incubated with the cells for 24 h.

Images of the scratched area were captured at 0 h (immediately after scratch formation) and after 24 h of treatment using a Nikon Eclipse Ni-U upright light microscope (Nikon Corporation, Tokyo, Japan) equipped with a Nikon Digital Sight 10 high-resolution camera (Nikon Corporation, Tokyo, Japan). Wound closure was quantified using ImageJ software by measuring the scratch area at both time points. The percentage of wound closure was calculated using the formula [29]:

$$Wound\;Closure\;\left(\%\right)=\left(\frac{A_{0}-A_{24}}{A_{0}}\right)\times 100$$

where *A*_0_ is the scratch area at time zero, and *A*_24_ is the area after 24 h.

All experiments were performed in triplicate (*n* = 3), and results were expressed as mean ± SD.

### 3.10. Statistical Analysis

All experiments were performed in triplicate, and results are expressed as mean ± standard deviation (SD). Statistical differences between groups were assessed using one-way analysis of variance (ANOVA), followed by Tukey’s post hoc test for multiple comparisons. A *p*-value of less than 0.05 was considered statistically significant. Data analysis was carried out using GraphPad Prism version 8.0 (GraphPad Software, San Diego, CA, USA).

## 4. Results

### 4.1. Characterization of ZnO NPs

#### 4.1.1. Dynamic Light Scattering and Zeta Potential Analysis

Prior to ball milling, ZnO NPs exhibited a mean size greater than 5 µm, confirming their initial microscale nature. Following ball milling and dispersion in 3% *w/v* PVA solution, the ZnO nanosuspension exhibited a mean hydrodynamic diameter of 117 ± 36 nm, indicating effective size reduction into the nanoscale range. ZnO NPs have been reported to exhibit enhanced antibacterial activity, highlighting the relevance of particle size in determining biological performance [19]. The PDI was 0.30, reflecting moderate size distribution homogeneity. The ZnO NPs exhibited a zeta potential of −9.03 ± 1.5 mV, indicating moderate colloidal stability. The negative charge is primarily attributed to the deprotonation of surface hydroxyl groups at pH values below ZnO’s isoelectric point (~9–10), a behavior commonly observed in aqueous systems [30]. Additionally, the PVA solution provides steric stabilization through its hydroxyl-rich backbone, preventing nanoparticle aggregation without substantially altering the native surface charge [31]. The moderately negative zeta potential supports the stable dispersion of ZnO NPs within the polymeric matrix, which is critical for homogeneous incorporation into electrospun fibers and for ensuring controlled antibacterial release behavior [32]. Comparable hydrodynamic diameters and moderately negative zeta potential values have been reported for ZnO nanosuspensions incorporated into electrospun PVA-based wound dressing systems, where such colloidal characteristics were shown to favor homogeneous nanoparticle distribution and reproducible antibacterial performance [33].

#### 4.1.2. Gravimetric Analysis of ZnO NPs

To determine the concentration of the ZnO NPs in the final suspension, gravimetric analysis was performed. Although 4% *w*/*v* ZnO NPs were initially added to the PVA solution, the final concentration was determined to be 3.04% *w*/*v*. This reduction is likely due to processing losses during ball milling.

### 4.2. Characterization of Electrospun Nanofibers

#### 4.2.1. Fiber Morphology

**Figure 1:**
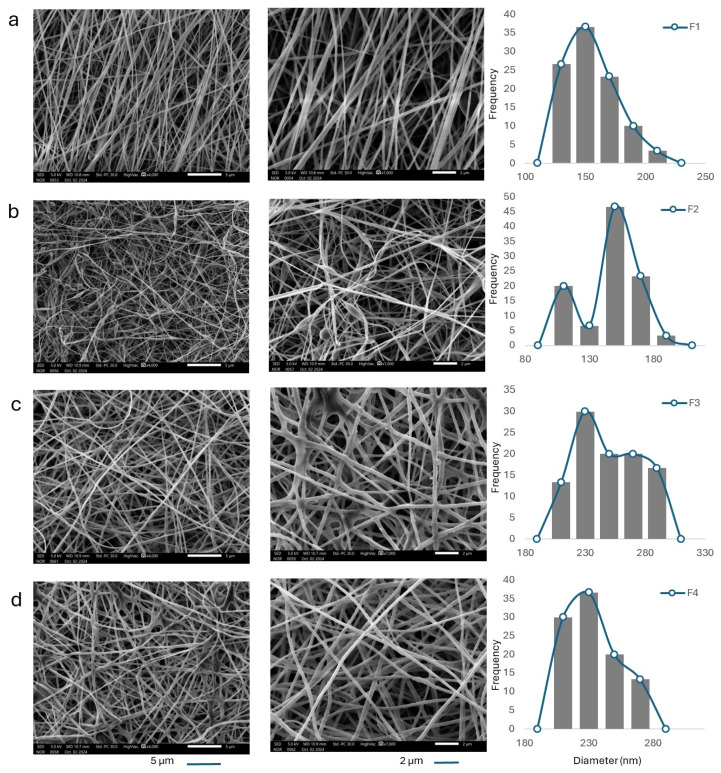
SEM images and the corresponding fiber-diameter distribution histograms of electrospun nanofiber formulations: (**a**) F1 (blank PVA nanofibers), (**b**) F2 (PVA/ZnO nanofibers), (**c**) F3 (PVA/ATR nanofibers), (**d**) F4 (PVA/ATR/ZnO nanofibers). Scale bars represent 5 µm (low magnification) and 2 µm (high magnification).

The fiber morphology and diameter distribution of the electrospun scaffolds were analyzed using SEM and imageJ analysis software. SEM micrographs (Figure 1) confirmed that all electrospun scaffolds exhibited continuous, bead-free nanofiber networks, indicating stable electrospinning conditions. The blank PVA nanofibers (F1) exhibited an average diameter of 156.03 ± 22.65 nm. The incorporation of ZnO NPs into the PVA matrix in F2 led to a slight reduction in the average fiber diameter to 147.65 ± 20.66 nm. This reduction might be attributed to the increased electrical conductivity of the spinning solution in the presence of the negatively charged ZnO NPs, which could enhance electrostatic stretching of the polymer jet and result in thinner fibers during electrospinning [34]. Similar ZnO-driven reductions in fiber diameter, attributed to increased solution conductivity and enhanced jet stretching, have been reported in electrospun PVA-based wound dressing systems incorporating ZnO nanoparticles, confirming the role of metal-oxide fillers in modulating electrospinning dynamics and fiber architecture [35].

In contrast, loading the polymer matrix with ATR in F3 produced a significant increase in fiber diameter to 246.40 ± 24.07 nm. This effect is likely due to ATR–PVA interactions that disrupt intermolecular hydrogen bonding, thereby reducing polymer entanglement and increasing jet thickness during electrospinning. This behavior aligns with prior reports demonstrating that hydrophobic drug incorporation into hydrophilic polymers can alter solution viscoelasticity and yield thicker fibers [26]. F4 fibers exhibited a diameter of 233.07 ± 20.78 nm indicating that while ZnO partially counteracts ATR-induced thickening through conductivity effects, ATR remains the dominant factor influencing fiber morphology. Notably, no nanoparticle aggregation or surface crystallization was observed, suggesting the uniform dispersion of both ATR and ZnO NPs within the fiber matrix. These morphological observations are consistent with DSC findings showing reduced crystallinity and strong polymer–nanoparticle interaction [26].

Statistical analysis using one-way ANOVA followed by Tukey’s post hoc test showed no significant difference between F1 and F2 (*p* = 0.2326), whereas ATR-containing formulations (F3 and F4) exhibited significantly larger fiber diameters than F1 and F2 (*p* < 0.0001). A significant difference was also observed between F3 and F4 (*p* = 0.0152).

#### 4.2.2. DSC Thermograms

**Figure 2:**
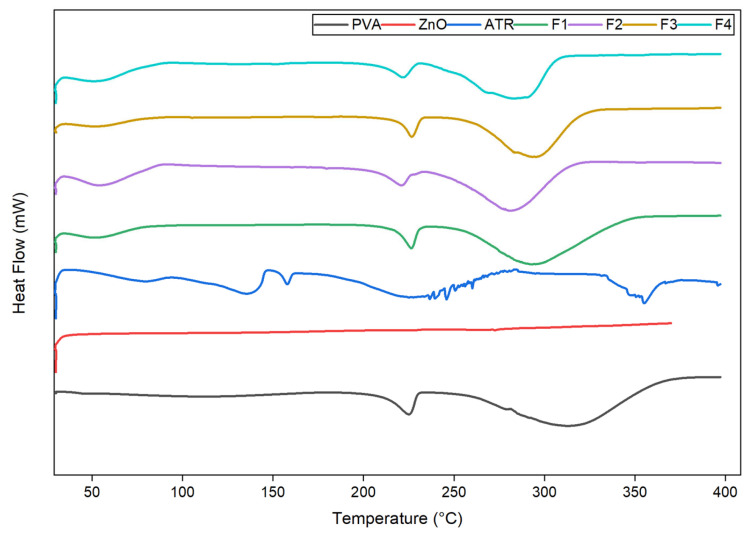
DSC thermogram of individual components (PVA, ZnO, ATR), and F1 (blank PVA nanofibers), F2 (PVA/ZnO nanofibers), F3 (PVA/ATR nanofibers), F4 (PVA/ATR/ZnO nanofibers).

The DSC thermograms (Figure 2) revealed distinct thermal transitions for the pure components and electrospun nanofiber formulations. Pure ATR exhibited two endothermic peaks at approximately 93 °C (dehydration) and 152 °C (ΔH = 109 J g^−1^), confirming its crystalline nature and aligning with the reported melting range of 155–160 °C followed by decomposition near 235 °C [36]. ZnO NPs showed a nearly featureless profile with only a weak deviation around 300 °C, demonstrating high thermal stability and confirming their inertness within the electrospinning range [37]. The pure PVA, blank PVA nanofibers (F1) showed endothermic peaks at approximately ≈ 90 °C (moisture loss), 226 °C (melting), and 294 °C (degradation), consistent with the semi-crystalline structure of PVA [38]. Incorporation of ZnO NPs (F2) reduced the melting enthalpy from ≈62 J g^−1^ to ≈30 J g^−1^ and slightly shifted the melting point to 221 °C, indicating restricted polymer chain mobility and reduced crystallinity due to ZnO–PVA interactions [39].

In ATR-loaded fibers (F3), the absence of the characteristic ATR melting endotherm at 155–160 °C confirmed drug amorphization and molecular dispersion within the polymeric network, likely facilitated by rapid solvent evaporation during electrospinning [40]. In the dual-loaded formulation (F4), a melting endotherm corresponding to the PVA matrix was observed at approximately 222 °C, followed by thermal degradation at approximately 284 °C, accompanied by a reduced fusion enthalpy (≈35 J g^−1^). This reduction in ΔH relative to F1 and F2 is consistent with decreased polymer crystallinity in the presence of both ATR and ZnO NPs. These findings indicate good component compatibility, stable amorphous incorporation of ATR, and a thermally robust PVA/ZnO matrix, which may be advantageous for formulation stability and controlled drug delivery applications [39].

Comparable reductions in polymer crystallinity and suppression of drug melting endotherms have been reported in electrospun PVA-based nanofiber systems incorporating ZnO nanoparticles and hydrophobic bioactive agents for wound healing applications. Kenawy et al. reported that ZnO incorporation into electrospun PVA-based nanofibers led to decreased melting enthalpy and restricted polymer chain mobility, without compromising thermal integrity, supporting the interpretation that nanoparticle incorporation disrupts polymer packing while maintaining functional stability [41].

#### 4.2.3. FTIR Spectra

**Figure 3:**
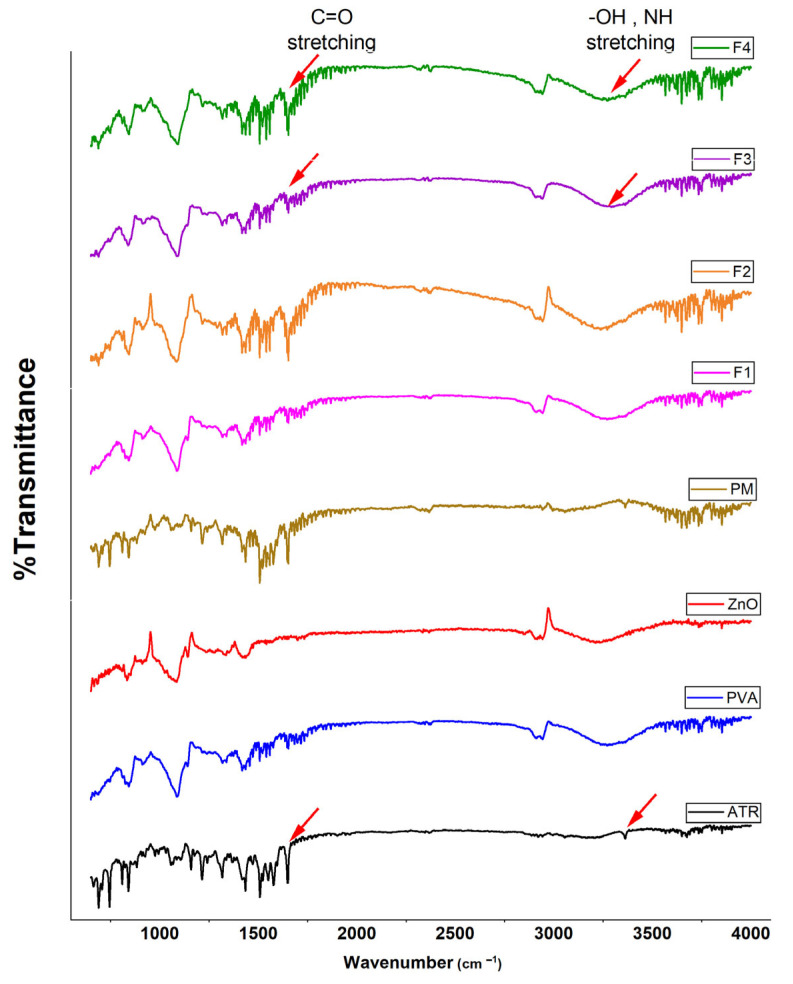
FTIR spectra of individual components (PVA, ZnO, ATR), PM, F1 (blank PVA nanofibers), F2 (PVA/ZnO nanofibers), F3 (PVA/ATR nanofibers), and F4 (PVA/ATR/ZnO nanofibers).

The FTIR spectra of pure components (PVA, ZnO, and ATR), physical mixture (PM), and electrospun formulations (F1–F4) are shown in Figure 3. The spectrum of PVA exhibited characteristic bands, including a broad O–H stretching vibration around 3270 cm^−1^ and a peak near 1140 cm^−1^ attributed to C–O stretching in the crystalline domains. ZnO displayed a distinct but weak absorption in the low wavenumber region ~450 cm^−1^, corresponding to Zn–O stretching vibrations, although this peak is generally weak due to the inorganic nature of the material.

ATR showed multiple sharp peaks, notably those corresponding to O–H and N–H stretching vibrations 3300–3200 cm^−1^, as well as prominent carbonyl (C=O) stretching near 1700 cm^−1^, reflecting its complex molecular structure. These peaks were largely retained in the PM, confirming the absence of interactions prior to processing. However, in the electrospun formulations, particularly F3 and F4, several spectral changes were observed. F1 (blank PVA) maintained the characteristic peaks of PVA, suggesting that the electrospinning process did not chemically alter the polymer. In F2, where ZnO was incorporated, a reduction in the intensity of the Zn–O peak was observed, possibly due to its dispersion within the fiber matrix. F3, containing ATR, showed broader and less intense O–H (~3270 cm^−1^) and C=O (~1700 cm^−1^) bands compared to the pure ATR, indicating possible hydrogen bonding and interaction between ATR and PVA chains during electrospinning. These changes may also suggest partial drug amorphization. In F4, which combines both ATR and ZnO NPs, further broadening and peak shifts were evident, accompanied by attenuation of characteristic ATR and ZnO signals. These spectral modifications support the hypothesis of intermolecular interactions among polymer, drug, and nanoparticles, resulting in a more integrated fiber network. Similar FTIR band broadening and attenuation phenomena have been reported in electrospun PVA-based wound dressing systems containing ZnO nanoparticles, where such spectral changes were attributed to polymer–nanoparticle interfacial interactions, primarily hydrogen bonding, supporting interaction-driven integration within the fibrous matrix [42].

#### 4.2.4. XRD Patterns

**Figure 4:**
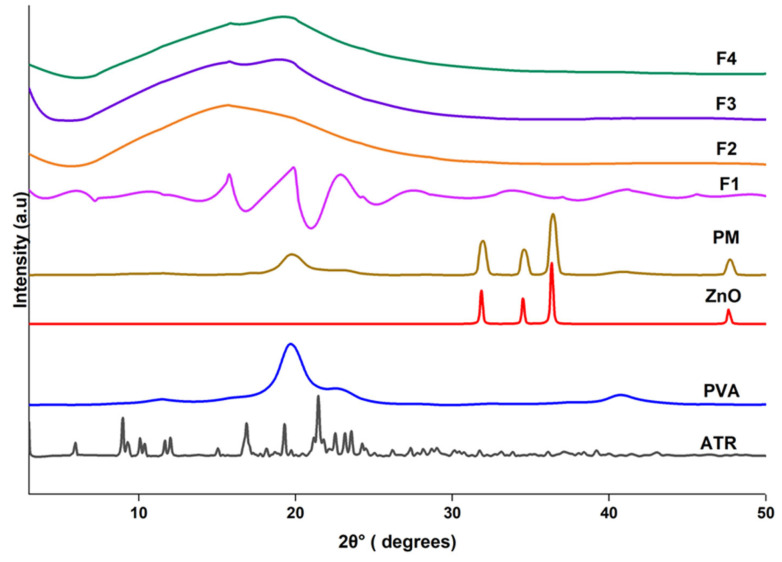
XRD patterns of individual components (PVA, ZnO, ATR), PM, F1 (blank PVA nanofibers), F2 (PVA/ZnO nanofibers), F3 (PVA/ATR nanofibers), and F4 (PVA/ATR/ZnO nanofibers).

XRD analysis was conducted to assess the physical state and potential interactions among the components within the nanofiber formulations (Figure 4). The pure ATR displayed numerous sharp and intense diffraction peaks indicative of its highly crystalline nature. ZnO NPs similarly exhibited distinct, sharp peaks at characteristic 2θ values of approximately 31.8°, 34.5° and 36.3°, consistent with its hexagonal wurtzite crystal structure, confirming its crystalline identity. In contrast, PVA exhibited a broad halo centered around 19.5°, suggesting its semi-crystalline structure.

The PM retained some of the characteristic peaks of both ATR and ZnO, although slightly reduced in intensity, indicating limited interaction between components when simply blended. The XRD pattern of the blank nanofibers (F1) showed the characteristic broad peak of PVA, confirming fiber formation without any added actives. In F2 (ZnO-loaded fibers), the characteristic crystalline peaks of ZnO disappeared, indicating either molecular dispersion or a reduction in crystallinity due to the electrospinning process. Similar attenuation/weak appearance of ZnO reflections in electrospun PVA-based matrices has been reported and is commonly attributed to low nanoparticle loading, strong embedding within the polymer network, and masking by the dominant amorphous polymer halo rather than complete loss of ZnO crystallinity [41].

Notably, in F3 (ATR-loaded fibers), the disappearance of ATR sharp crystalline peaks suggests successful encapsulation of the drug in an amorphous or molecularly dispersed state within the PVA matrix. This amorphization could enhance the solubility and bioavailability of ATR. In F4 (ATR/ZnO-loaded fibers), the absence of sharp diffraction peaks from both ATR and ZnO crystalline peaks indicates that both components were effectively dispersed in the amorphous state, likely facilitated by the electrospinning process and interactions with the polymeric matrix. These observations confirm that the formulation strategy successfully transformed the active agents into an amorphous form or dispersed incorporation, which may contribute to improved dissolution behavior and biological performance.

### 4.3. DL% and EE% Measurements

The DL% and EE% of ATR in the nanofiber formulations were determined for both F3 and F4. The F3 formulation exhibited a drug loading of 9.01 ± 0.26% with an EE% of 99.13 ± 3.01%, indicating nearly complete incorporation of the drug into the nanofibrous matrix. In comparison, the F4 formulation, which contained 9% ZnO NPs by weight, demonstrated a slightly lower drug loading of 7.68 ± 0.43% and an EE% of 89.16 ± 5.09%.

The high entrapment efficiency observed in the F3 nanofibers is likely due to the miscibility of methanol and water, which enables uniform drug distribution during electrospinning. These findings are consistent with prior studies reporting efficient drug encapsulation in hydrophilic polymeric nanofibers using electrospinning, particularly when drugs are molecularly dispersed in the spinning solution [43,44]. Similar near-complete entrapment efficiencies have been reported for atorvastatin and other poorly water-soluble drugs incorporated into electrospun PVA-based nanofibers, where homogeneous drug distribution within the spinning solution enabled efficient retention during fiber formation [26].

In the F4 formulation, the slight reduction in DL% and EE% may be attributed to the presence of ZnO NPs, which can alter the viscosity, conductivity, and molecular interactions within the spinning solution. This may lead to partial drug expulsion during fiber formation or non-uniform distribution [45]. Previous electrospinning studies have shown that incorporation of inorganic nanoparticles can modify solution conductivity and jet stability, resulting in redistribution of dissolved drugs and a modest reduction in drug entrapment efficiency [46]. This competition between drug molecules and ZnO NPs for interaction sites within the polymer matrix may reduce EE. Such competition is influenced by physisorption mechanisms, including electrostatic and van der Waals forces, as previously reported in nanofiber composites incorporating charged nanoparticles. Such non-covalent interactions have been widely reported to govern drug retention in nanofiber–nanoparticle composite systems and are sufficient to influence loading efficiency without compromising overall formulation performance still reflecting effective drug incorporation suitable for therapeutic applications [47,48].

Maintaining high drug loading and EE% is critical for ensuring controlled release performance and minimizing material waste. The results confirm that both formulations are pharmaceutically acceptable, with F3 showing slightly superior drug retention.

### 4.4. In Vitro Drug Release

**Figure 5:**
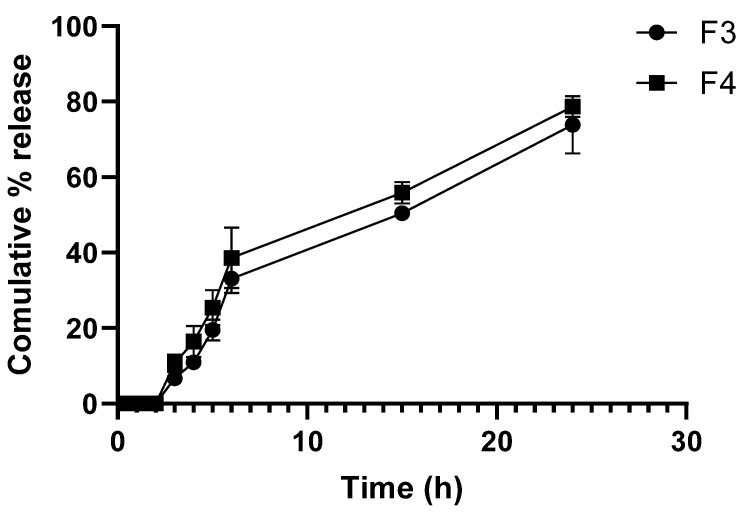
Cumulative in vitro release profiles of ATR from F3 (PVA/ATR nanofibers), and F4 (PVA/ATR/ZnO nanofibers) over 24 h. Data are expressed as mean ± SD (*n* = 3).

The cumulative drug release profiles of ATR from both the F3 and F4 were evaluated over a 24 h period in an ethanol: PBS (2:3 *v*/*v*) medium at 37 °C (Figure 5). At the early time points (up to 2 h), F3 formulation exhibited minimal release, with a clear increase starting at the 3 h mark. In contrast, F4 formulation showed a similar initial delay, followed by a more sustained release over time. After 24 h, F3 fibers released approximately 73.8% ± 7.6 of their drug content, while the F4 fibers released 78.7% ± 2.8, indicating a slightly enhanced release in the presence of ZnO.

While both formulations exhibited limited burst release during the initial phase, the incorporation of ZnO NPs in F4 was associated with a more uniform and extended-release profile, particularly between 3 and 6 h, during which higher cumulative release was observed compared with F3.

The drug release behavior observed in both formulations is characteristic of diffusion-controlled release from hydrophilic polymeric matrices such as PVA, which swells upon contact with aqueous media, allowing gradual drug diffusion over time [49]. The initial lag phase is likely due to the slow hydration and swelling of the nanofibers before noticeable release begins.

The incorporation of ZnO NPs in F4 formulation appeared to slightly enhance the release rate, particularly in the mid-phase (3–6 h). This may be attributed to changes in fiber structure or porosity resulting from ZnO NPs dispersion, which can facilitate faster water penetration and matrix relaxation [50]. Additionally, ZnO NPs have been reported to act as a pore-forming agent, potentially increasing diffusion channels within the nanofiber matrix [47,50]. Comparable diffusion-controlled and sustained drug release profiles have been reported for ZnO-containing electrospun PVA-based wound dressing matrices, where incorporation of ZnO nanoparticles was shown to modify the polymer matrix structure and swelling behavior, thereby influencing release kinetics in a manner consistent with the trends observed in the present study [48].

The absence of a sharp burst release is favorable for wound healing applications, as it indicates a controlled and sustained delivery profile. Sustained release of ATR is particularly important, as its anti-inflammatory, pro-angiogenic, and antibacterial activities are more effective when maintained over an extended period [51]. These findings are consistent with previous reports where polymer–nanoparticle composite systems were shown to prolong release kinetics and improve drug stability and performance at the site of application [52].

Although PVA is intrinsically water-soluble, the electrospinning process produces a physically stabilized fibrous network characterized by rapid solvent evaporation, polymer chain alignment, and extensive intermolecular hydrogen bonding, which collectively promote partial crystallization and reduced chain mobility [53]. These structural features markedly delay immediate dissolution and enable the nanofibrous scaffold to maintain integrity upon exposure to aqueous media. In addition, the aqueous stability of the scaffold is further supported by the high degree of hydrolysis of the PVA used in this study (98%), as highly hydrolyzed PVA contains a greater density of hydroxyl groups that enhance intermolecular hydrogen bonding, promote crystallite formation, and reduce water solubility compared with partially hydrolyzed grades [54]. Consequently, electrospun fibers prepared from highly hydrolyzed PVA can retain structural integrity for extended periods in aqueous environments before complete dissolution. Similar delayed-dissolution behavior and aqueous stability have been widely reported for electrospun PVA-based wound-dressing systems, where fiber morphology and crystallinity govern scaffold persistence rather than bulk polymer solubility [55,56].

### 4.5. Antibacterial Activity

#### 4.5.1. MIC Analysis

The antibacterial activity of free ATR, free ZnO, and their combination was first evaluated to establish the intrinsic antibacterial potency of the individual agents and their combined use (Table 2). MIC values for free agents are expressed in µg/mL. Free ATR and free ZnO NPs demonstrated antibacterial activity primarily against Gram-positive strains, with lower MIC values observed for *S. aureus* and *S. epidermidis* than for MRSA, consistent with previous reports on statin- and ZnO-based antibacterial activity [4,19,57]. The combination of ATR and ZnO NPs resulted in substantially lower MIC values against all Gram-positive strains than the individual agents, indicating enhanced antibacterial activity. In contrast, all free drug treatments exhibited limited activity against *P. aeruginosa*, with MIC values remaining high.

The antibacterial performance of the electrospun nanofiber formulations was evaluated to assess the effect of drug incorporation into the fibrous delivery system (Table 3). MIC values for nanofiber formulations are expressed as fiber mass equivalents (mg/mL) based on nanofiber extracts and are therefore not directly comparable to free drug MICs. Blank PVA nanofibers (F1) showed no antibacterial activity against any tested strain. Single-agent nanofibers containing ZnO NPs (F2) or ATR (F3) exhibited antibacterial activity against Gram-positive bacteria, with moderate MIC values, while both formulations remained inactive against *P. aeruginosa*.

The dual-loaded nanofiber formulation (F4) demonstrated the lowest MIC values among all fiber formulations against Gram-positive strains, with MICs of 1.5 mg/mL for *S. aureus* and *S. epidermidis* and 2.0 mg/mL for MRSA. This indicates enhanced antibacterial activity of the co-loaded system relative to single-agent nanofibers. Against *P. aeruginosa*, F4 exhibited limited activity, consistent with the reduced susceptibility observed for free agents. Collectively, these results demonstrate that co-loading ATR and ZnO NPs into electrospun nanofibers improves antibacterial performance against Gram-positive bacteria compared with single-agent fiber systems, while Gram-negative activity remains limited.

Although higher mass-equivalent concentrations of nanofibers were required to achieve inhibitory endpoints compared with free agents, this reflects differences in how concentrations are defined and delivered in the two systems. Free-drug MICs are expressed as µg/mL of active compound, whereas nanofiber MICs are reported as mg/mL fiber equivalents that include a substantial fraction of inactive polymer. Moreover, only a portion of the loaded ATR and ZnO NPs are expected to become bioavailable in the broth during the extraction/incubation period due to matrix retention, incomplete extraction, and particulate behavior of ZnO. Therefore, higher fiber mass is required to provide sufficient accessible ATR and ZnO NPs at the bacterial interface, even when the total loading ratio in the formulation is maintained.

#### 4.5.2. Bacterial Growth Inhibition

**Figure 6:**
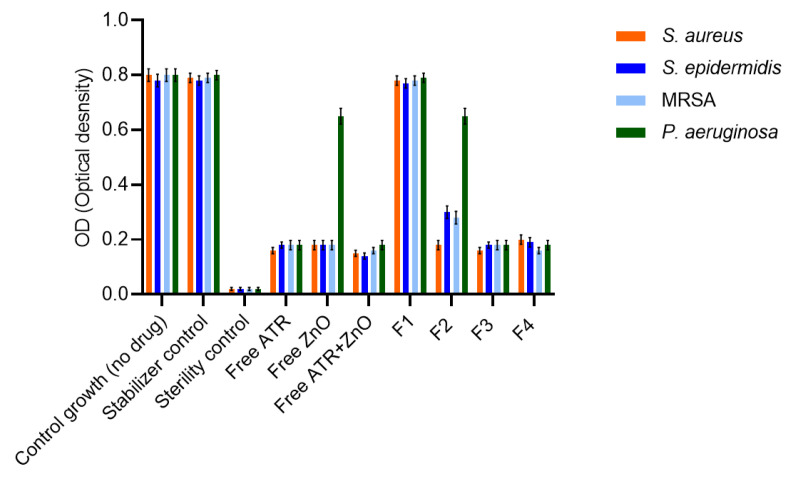
OD600 measurements after 24 h incubation for free drugs, nanofiber formulations, and controls at MIC-relevant concentrations. OD_net_ values are presented as mean ± SD.

Bacterial growth inhibition was further evaluated by measuring OD600 after 24 h incubation at the corresponding MIC for each treatment (Figure 6). For all Gram-positive strains, treatments that exhibited lower MIC values also resulted in lower OD_net_ values, supporting the MIC findings. Free ATR and free ZnO NPs reduced bacterial growth individually, while the ATR and ZnO NPs combination produced further reductions in OD_net_ values for *S. aureus*, *S. epidermidis*, and MRSA.

Among the nanofiber formulations, F4 consistently reduced bacterial growth to OD_net_ values ≤ 0.20 for all Gram-positive strains, whereas single-agent nanofibers (F2 and F3) showed higher OD_net_ values. Blank nanofibers (F1) did not inhibit bacterial growth. For *P. aeruginosa*, higher OD_net_ values were observed across all treatments, indicating a significantly reduced susceptibility and limited growth inhibition (*p* < 0.0001), consistent with the MIC results. Vehicle controls and stabilizer controls did not affect bacterial growth, and sterility controls remained free of contamination, confirming the validity of the assay conditions.

#### 4.5.3. Synergy Analysis (FICI)

Synergistic interactions between ATR and ZnO NPs were quantified using the checkerboard assay and expressed as FICI values (Table 4). For free agents, FICI analysis demonstrated synergistic interactions against *S. aureus*, *S. epidermidis*, and MRSA, while no synergistic interaction was observed against *P. aeruginosa*, consistent with previous reports on ZnO-based combination antibacterial systems [58]. The requirement for relatively high ZnO NPs concentrations (MICs exceeding 1 µg/mL) to inhibit *P. aeruginosa* likely reflects the inherent resistance of Gram-negative bacteria and the limited penetration of ZnO NPs through the outer membrane, consistent with high MIC values reported for ZnO NPs in previous studies [59].

The antibacterial evaluation demonstrated that co-loading ATR and ZnO NPs into electrospun PVA nanofibers enhanced antibacterial performance against Gram-positive bacteria compared with single-agent fiber formulations. This improvement was evident from reduced MIC values and lower OD_net_ measurements for the dual-loaded formulation (F4), particularly against *S. aureus*, *S. epidermidis*, and MRSA. Importantly, the antibacterial trends observed for nanofiber formulations were consistent with those obtained for free ATR and ZnO NPs combinations, indicating that incorporation into the fibrous matrix preserved the intrinsic antibacterial activity of both agents. The enhanced antibacterial performance of F4 relative to single-agent fibers can be attributed to the simultaneous local delivery of ATR and ZnO NPs at the bacterial interface. While ATR-loaded (F3) and ZnO-loaded (F2) nanofibers individually inhibited Gram-positive bacteria, their co-delivery resulted in lower MIC values, indicating additive or synergistic effects depending on the bacterial strain. This strain-dependent behavior is consistent with previous observations of statin-associated antibacterial activity and ZnO-mediated enhancement [7,16,18].

In contrast, all formulations exhibited limited antibacterial activity against *P. aeruginosa*, regardless of whether ATR and ZnO NPs were delivered as free agents or within nanofibers. This reduced susceptibility is consistent with the intrinsic resistance of Gram-negative bacteria, which possess an outer membrane rich in lipopolysaccharides that restricts the penetration of hydrophobic compounds and limit nanoparticle–cell interactions [60]. In addition, effective inhibition of *P. aeruginosa* by ZnO has been reported to require relatively high concentrations, which may not be achievable or practical within electrospun fiber formulations [59].

The nanofiber platform offers distinct advantages beyond intrinsic antibacterial potency. Electrospun fibers provide a high surface-area-to-volume ratio and intimate contact with bacterial cells, facilitating localized delivery and sustained exposure of antibacterial agents. The absence of antibacterial activity in blank fibers confirmed that the polymer matrix itself did not contribute to bacterial inhibition, while the maintained activity of drug-loaded fibers demonstrates that electrospinning did not compromise drug function [10].

### 4.6. In Vitro Cytotoxicity Assay

**Figure 7:**
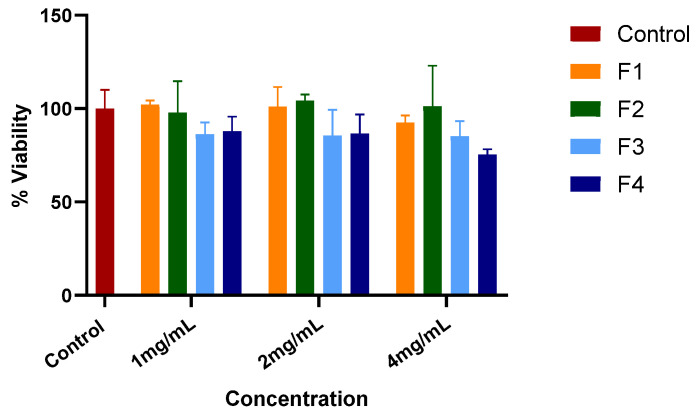
Cytotoxicity of electrospun nanofiber extracts (F1–F4) evaluated on hFFs after 24 h exposure using an MTT assay.

The cytotoxicity of the electrospun nanofiber formulations (F1–F4) was assessed using an MTT assay on hFFs following 24 h exposure (Figure 7). At a concentration of 1 mg/mL, all formulations maintained high cell viability, with values of 102.12 ± 2.17% (F1), 88.65 ± 22.91% (F2), 86.21 ± 6.38% (F3), and 87.82 ± 7.89% (F4). These results indicate that the incorporation of ATR and ZnO nanoparticles did not induce cytotoxic effects. At a 2 mg/mL concentration, F1 and F2 maintained 100% viability, suggesting that the PVA matrix and ZnO-loaded formulation were well tolerated. F3 and F4 showed moderate reductions in viability (85.54 ± 13.81% and 86.55 ± 10.24%, respectively), which may reflect concentration-dependent cellular responses to ATR exposure. However, viability remained well above the 70% threshold commonly used to define cytocompatibility.

At the highest tested concentration (4 mg/mL), F1 and F2 continued to demonstrate high viability (92.49 ± 3.81% and 101.24 ± 21.75%, respectively), confirming the biocompatible nature of the PVA matrix and ZnO incorporation at the tested levels. F3 maintained viability at 85.17 ± 8.09%, indicating good tolerance of ATR release. A more pronounced reduction was observed for the dual-loaded formulation (F4), which exhibited 75.49 ± 2.74% viability. Although this represents the lowest viability among tested groups, it remained above the ISO 10993-5 cytotoxicity threshold (≥70%), supporting acceptable cytocompatibility even at the highest extract concentration [61].

The slightly reduced viability observed for F4 at elevated concentration may result from the combined biological activity of ATR and ZnO, potentially leading to enhanced oxidative or membrane-associated cellular stress. Nevertheless, the absence of severe cytotoxicity suggests that the controlled release from the electrospun matrix mitigates acute exposure effects.

### 4.7. In Vitro Wound Healing Activity

**Figure 8:**
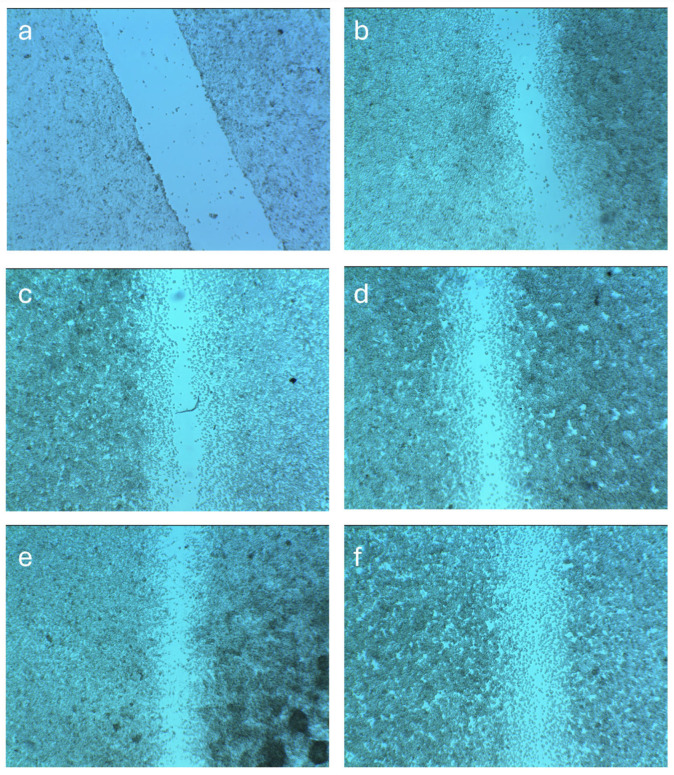
Representative images of in vitro wound healing (scratch assay) in hFF cultures. (**a**) Control at 0 h; (**b**) Control at 24 h; (**c**) F1 (blank PVA nanofiber); (**d**) F2 (PVA/ZnO nanofibers); (**e**) F3 (PVA/ATR nanofibers); (**f**) F4 (PVA/ATR/ZnO nanofibers). Wound closure was assessed after 24 h of treatment.

The wound healing potential of the developed formulations was assessed using an in vitro scratch assay on hFF cells, and the migration of cells into the wound area was monitored after 24 h of treatment. As shown in Figure 8, all nanofiber treatments significantly enhanced wound closure compared with the untreated control (*p* ≤ 0.0295), with the dual-loaded formulation (F4) showing the greatest improvement (*p* < 0.0001 vs. control). Untreated control cells exhibited only partial closure of the scratch area, with 58.20 ± 8.11% closure, reflecting the baseline migratory capacity of the cells under standard conditions. Treatment with F1 slightly improved healing (72.99 ± 6.62%), likely due to the moist environment and biocompatible nature of the fibers, which facilitate cell migration. Similarly, F2 fiber further enhanced wound closure to 76.71 ± 2.98%, indicating the beneficial role of ZnO in promoting tissue regeneration, possibly through its reported antibacterial and anti-inflammatory activities that help maintain a favorable healing environment [62,63]. The incorporation of ATR into the formulation demonstrated even more pronounced healing activity (83.58 ± 1.04%), consistent with previous findings that ATR promotes angiogenesis, enhances fibroblast proliferation, and modulates inflammation during wound repair [64,65].

The dual-loaded F4 nanofiber exhibited the highest closure percentage (92.41 ± 2.36%). Comparisons among formulations indicated that F4 achieved significantly greater wound closure than F1 and F2 (*p* = 0.0053 and 0.0209, respectively), while differences between F2 and F3 and between F3 and F4 were not statistically significant (*p* > 0.05).

These findings suggest that both ATR and ZnO, when delivered via nanofibrous scaffolds, can facilitate cellular behaviors conducive to wound healing, particularly migration. ATR is known to promote vascular endothelial growth factor (VEGF) expression and fibroblast proliferation [66]. Meanwhile, ZnO NPs have been widely reported to stimulate wound repair by enhancing re-epithelialization, collagen deposition, and reactive oxygen species-mediated cell signaling [21,67,68]. The combined effects in F4 likely stem from the synergistic antibacterial and pro-regenerative properties of both agents, coupled with the structural benefits of the nanofiber matrix, which mimics the native extracellular matrix and provides a conducive environment for cell proliferation and migration. These results support the proposed multifunctional role of the nanofibrous system as both an antibacterial dressing and a wound healing enhancer. The observed wound closure may be attributed to the combined effects of ATR-mediated upregulation of VEGF and ZnO’s antibacterial and ROS-scavenging properties [69,70].

## 5. Conclusions

This study successfully developed a novel multifunctional localized platform by co-loading ATR and ZnO NPs into electrospun PVA nanofibers. Morphological and physicochemical characterization confirmed that both active agents were effectively integrated into a stable, bead-free fibrous network, preserving their intrinsic biological properties while achieving a sustained release profile suitable for wound care.

The dual-loaded nanofiber system (F4) exhibited superior antibacterial performance against Gram-positive bacteria—including *S. aureus*, *S. epidermidis*, and MRSA—compared to single-agent formulations. While activity against *P. aeruginosa* remained limited due to the intrinsic resistance of Gram-negative outer membranes, the platform’s primary strength lies in its dual-action capability.

Beyond infection control, the F4 nanofibers achieved the highest rate of in vitro wound closure, significantly outperforming blank and single-drug fibers. This enhancement is attributed to the combined pro-migratory effects of ATR and the regenerative properties of ZnO NPs, supported by a nanofiber matrix that mimics the natural extracellular matrix. These findings position ATR/ZnO co-loaded nanofibers as a highly effective, biocompatible localized treatment for infected wounds. Future research should focus on modifying the polymer matrix to expand the spectrum of activity toward Gram-negative pathogens and conducting in vivo studies to validate the safety and efficacy of this platform in complex wound environments.

## Figures and Tables

**Table 1:** Composition and encapsulation characteristics of electrospun PVA nanofiber formulations (F1–F4), including blank and ATR and/or ZnO NP-loaded fibers.

Formulation	ZnO NPs *	ATR *	PVA *
F1	-	-	100%
F2	9%	-	91%
F3	-	9%	91%
F4	7%	9%	84%

* The percentages represent the actual mass fractions of each component relative to the total solid content in the electrospinning solution.

**Table 2:** MICs of free ATR, ZnO, and ATR/ZnO against Gram-positive and Gram-negative bacterial strains determined by the broth microdilution method after 24 h incubation. MIC values for free agents are expressed in µg/mL.

Formulation	*S. aureus*	*S. epidermidis*	MRSA	*P. aeruginosa*
Free ATR	92 µg/mL	92 µg/mL	184 µg/mL	184 µg/mL
Free ZnO	72 µg/mL	72 µg/mL	144 µg/mL	>144 µg/mL
Free ATR/ZnO	23/18 µg/mL	23/18 µg/mL	46/36 µg/mL	184/144 µg/mL

**Table 3:** MICs of electrospun nanofiber formulations against bacterial strains. MIC values are expressed as fiber mass equivalents (mg/mL) based on nanofiber extracts prepared at 4 mg/mL.

Formulation	*S. aureus*	*S. epidermidis*	MRSA	*P. aeruginosa*
F1	>4.0 mg/mL	>4.0 mg/mL	>4.0 mg/mL	>4.0 mg/mL
F2	2.0 mg/mL	2.0 mg/mL	3.0 mg/mL	>4.0 mg/mL
F3	3.0 mg/mL	3.0 mg/mL	4.0 mg/mL	>4.0 mg/mL
F4	1.5 mg/mL	1.5 mg/mL	2.0 mg/mL	4.0 mg/mL

**Table 4:** FICI values of ATR and ZnO tested as free agents against bacterial strains.

Bacterial Strain	ATR/ZnO MIC (µg/mL)	FICI	Interpretation
*S. aureus*	11.6 + 18.1	0.375	Synergy
*S. epidermidis*	5.8 + 18.1	0.313	Synergy
MRSA	23.1 + 36.25	0.375	Synergy
*P. aeruginosa*	92.5 + 290	>1.0	Indifferent

## Data Availability

Data is contained within the article.
